# The Comparison of the Efficacy and Safety of Fractional 1064 nm Nd:YAG Picosecond Laser and Fractional Q‐Switched 1064‐nm Nd:YAG Laser in the Improvement of Photoaging: A Split‐Face Study

**DOI:** 10.1111/jocd.70307

**Published:** 2025-07-01

**Authors:** Qiuyue Tang, Qilei Che, Yuhong Xie, Lufeng Liu, Yulian Gao, Zixuan Zhou, Pengyu Zhao, Qiang He, Lixia Xie, Wenju Wang, Qingbiao Wa

**Affiliations:** ^1^ Department of Cosmetology Center Chengdu Second People's Hospital Chengdu, Si Chuan China; ^2^ Department of Dermatology Chengdu Second People's Hospital Chengdu, Si Chuan China

**Keywords:** Nd:YAG laser, photoaging, picosecond laser, pores, skin texture, wrinkles

## Abstract

**Background:**

Facial photoaging is a complex biological process influenced by both internal and external factors. It is characterized by wrinkles, enlarged pores, and rough skin. Despite various clinical treatments, they often have limited effectiveness, significant side effects, and extended recovery times. There is a need for more effective and safer treatment options. Preliminary evidence suggests significant differences in mechanisms between fractional 1064 nm Nd:YAG picosecond laser (FxPico) and fractional Q‐switched 1064‐nm Nd:YAG laser (QSF‐Nd:YAG). Photothermal tissue modulation is achieved in the QSF‐Nd:YAG platform through holographic beam splitting, whereas the FxPico system's microlens arrays (MLA) enable dual‐mode action through concurrent photothermal and laser‐induced optical breakdown (LIOB) mechanisms. The purpose of this study was to conduct a split‐face controlled trial on the same subject, observing the therapeutic effects of both modalities.

**Methods:**

Thirty patients with photoaging underwent five treatment sessions at weeks 0, 2, 4, 6, and 8. Each patient received FxPico on one side of the face and QSF‐Nd:YAG on the other. Follow‐up evaluations were conducted at 1 and 3 months post‐treatment. Efficacy was assessed using VISIA quantitative analysis, reflectance confocal microscopy (RCM), and histological examination. Safety was evaluated based on adverse events and patient‐reported pain.

**Results:**

Among the patients following the completion of five treatment sessions exhibited remarkable improvement in terms of facial wrinkles, pores and skin texture from VISIA quantitative numerical assessment (*p* < 0.001). Semi‐quantitative assessments from reflectance confocal microscopy analysis also demonstrated a significant decrease (*p* < 0.05). There were no statistically significant differences observed in the reduction rates of wrinkles, pores, skin texture counts, semi‐quantitative scores, and the Global Aesthetic Improvement Scale scores (*p* > 0.05) between the FxPico and QSF‐Nd:YAG sides. Histological analysis revealed increased collagen formation and improved elastic fiber alignment in both treatment groups, which objectively validates the therapeutic efficacy of both laser modalities for photoaging. Adverse events were mild, with FxPico causing more pain (*p* < 0.0001).

**Conclusion:**

Both FxPico and QSF‐Nd:YAG lasers are effective and safe for improving photoaging, with no significant differences in clinical outcomes. FxPico may offer superior collagen stimulation, albeit with higher pain levels.

**Trial Registration:**

China Clinical Trial Registry: ChiCTR2200058566

## 1 Introduction

Facial photoaging represents a multifaceted biological phenomenon influenced by both intrinsic and extrinsic factors, including genetic predisposition and environmental influences [1]. Of particular significance is the cumulative damage incurred from prolonged exposure to ultraviolet radiation [2]. This intricate course of facial photoaging manifests in various undesirable manifestations, such as the emergence of wrinkles, alterations in pigmentation, enlargement of pores, and rough skin. These aesthetic concerns not only compromise one's physical appearance but also contribute to an increased psychological burden experienced by affected individuals [3].

A diverse array of therapeutic modalities has been employed to ameliorate the effects of photoaging, encompassing retinoids, antioxidants, chemical exfoliation, dermabrasion, cosmetic surgery, intense pulsed light, ultrasound, radiofrequency, and laser interventions [4]. However, the effectiveness of these interventions in mitigating photoaging remains suboptimal. Ablative laser treatments, in particular, inflict direct harm upon the epidermal layer, resulting in pronounced discomfort during the procedure, protracted postoperative convalescence, post‐inflammatory hyperpigmentation, as well as the potential for infection and other untoward reactions [5, 6]. Of particular concern is the heightened risk of post‐inflammatory hyperpigmentation in individuals with darker skin tones. Conversely, non‐ablative laser therapies, exhibiting the advantages of minimal downtime, limited adverse events, and negligible epidermal damage, hold great promise in the management of photodamaged skin. Based on these premises, the present study was devised.

The Q‐switched 1064‐nm Nd:YAG laser (QSF‐Nd:YAG) emits a planar beam through the utilization of a holographic array lens, meticulously divided into either 25 or 81 smaller beams, each possessing a consistent energy distribution. Particularly in a heightened energy state, this laser demonstrates increased efficacy in the stimulation of collagen and elastic fiber regeneration. Notably, the QSF‐Nd:YAG laser presents a distinctive ability to ameliorate pore dilation. Perhaps this efficacy can be attributed, at least in part, to the generation of collagen surrounding the follicles, as well as its direct influence on the follicular epithelium, consequently inducing structural modifications and pore constriction [5].

The fractional 1064 nm Nd:YAG picosecond laser (FxPico) represents a novel nonablative method of rejuvenation, initially developed for the purpose of tattoo removal and hyperpigmentation correction [7]. Currently, fractional picosecond lasers equipped with diffractive optical elements (DOE) or microlens arrays (MLA) have also been introduced. By utilizing an MLA, the fluence of the picosecond laser can be further augmented, as densely arranged micro‐lenses partition the picosecond pulses into columns characterized by high fluence. This results in the formation of focal areas with intensities of high fluence juxtaposed against a background of lower fluence. Numerous recent clinical studies have demonstrated the safety and efficacy of this technique in the treatment of photoaging, exhibiting minimal side effects and negligible downtime [8, 9].

In theory, both of these fractional nonablative laser modalities possess the ability to stimulate the regeneration of collagen. This study employed a randomized, split‐face clinical trial to evaluate and compare the safety and efficacy of FxPico and QSF‐Nd:YAG laser in the treatment of facial wrinkles and pores associated with photoaging.

## 2 Materials and Methods

### 2.1 Study Design

A total of 32 individuals presenting with photoaging‐related facial wrinkles and enlarged pores were included in this study. Signed informed consent was signed prior to treatment. The inclusion criteria encompassed both male and female participants aged between 40 and 60 years, with Fitzpatrick skin types III–IV. Exclusion criteria consisted of pregnancy or lactation, concurrent presence of other dermatological conditions (such as photodermatitis, dermatitis, a history of keloid formation, or infection in the treatment areas), as well as recent interventions involving botulinum toxin injections, dermal fillers, or any form of skin rejuvenation treatment within the past 6 months.

Each of the enrolled subjects underwent a series of five treatment sessions, with a 2‐week interval between each session. The facial halves of each participant were randomly assigned to either receive treatment with the picosecond 1064‐nm Nd:YAG laser or the QSF‐Nd:YAG laser. Follow‐up evaluations were conducted at 1 and 3 months post the final treatment session. After every treatment session, participants were required to document any observed side effects and note the time taken for healing in each treated facial half.

### 2.2 Treatment Procedures

Before the commencement of the therapeutic interventions, the subjects were administered a topical anesthetic of lidocaine cream for a duration of 40 min. The fractionated non‐ablative handpiece operating at a wavelength of 1064‐nm Nd:YAG is specifically known as “Resolve” (PicoWay, Candela, Weyland, USA). The pulse duration of this laser was 450 ps. The 1064 nm Resolve resolution handpieces emitted a series of fractionated pulses, producing a 10 × 10 square array of laser energy distributed evenly among 100 beamlets within a 6 mm region. The treatment parameters were two to three passes with a fluence ranging from 1.9 to 2.1 mJ/microbeam and a repetition rate of 6–8 Hz; the desired clinical endpoint was erythema and acicular purpura.

The QSF‐Nd:YAG (QX MAX, Fotona) laser, whereby the laser beam was passed through a lens, thereby pixelating the output into microbeams arranged in a 9 × 9 pixel grid. The pulse energy employed was set at 1.5–2.0 J/cm^2^, with a repetition rate of 2 Hz and a spot size of 6 mm; the desired clinical endpoint was erythema and minuscule bleeding points. In the post‐treatment phase, an ice pack was used immediately, recombinant human epidermal growth factor gel twice daily for 1 week, and mometasone furoate cream once daily for 1–3 days were applied. Additionally, strict avoidance of sun exposure was advised for a duration of 1 week.

### 2.3 Clinical Assessment

Professional facial digital photographs were taken using a VISIA‐CR camera (Canfield Scientific, Fairfield, NJ) from three distinct angles (0°, 45°, and −45°). The evaluation encompassed a comprehensive VISIA quantitative numerical assessment, focusing on parameters such as wrinkles, pores, and skin texture. Moreover, the top of the cheekbones, where specific points were marked on the face during baseline measurements, was subjected to in vivo reflectance confocal microscopy (RCM) (VivaScope 1500@, Lucid, Rochester, NY) to observe the dynamic changes occurring in the dermis and epidermis. Semi‐quantitative scores [10] of skin aging, as determined by three dermatologists who remained unaware of the treatment details, were calculated. To further gauge the aesthetic improvement, a 5‐point Global Aesthetic Improvement Scale (GAIS) was employed, with scores ranging from 0 (indicating worsening) to 4 (indicating significant improvement). This GAIS assessment was carried out by three independent physicians at the 3‐month mark following the treatment.

Participants were requested to provide their assessment of treatment satisfaction by rating it on a Likert satisfaction scale ranging from 1 to 5 [11]. This scale allowed them to express their level of satisfaction, with 1 indicating a state of utmost dissatisfaction and 5 representing a state of complete contentment. The pain levels were evaluated by means of a visual analog scale, which ranged from 0 (representing no pain or discomfort) to 10 (representing intolerable or the worst possible pain). Throughout the follow‐up visits, any adverse effects such as erythema, purpura, hyperpigmentation, or infection were strictly recorded.

### 2.4 In Vivo Human Histological Examination

Three individuals voluntarily biopsied in the affected regions, written consent was signed. All skin biopsy specimens, measuring approximately 0.5–1 cm in dimension, were acquired via surgical excision; the skin samples were subjected to formalin fixation and subsequently stained with hematoxylin and eosin, picrosirius red stain, and victoria blue stain. The stained samples were then observed utilizing an Olympus BX52 microscope (Olympus, Tokyo, Japan).

### 2.5 Statistical Analyses

The data were analyzed employing SPSS software (version 23.0; IBM, Armonk, NY). Statistical significance was determined by *p*‐values of *p < 0.05. Categorical variables were expressed as percentages, while numerical variables were presented as mean ± SD for data exhibiting normal distribution and as median (range) for data with non‐normal distribution. The VISIA quantitative numerical measurements of wrinkles, pores, and skin texture, and the semi‐quantitative scores derived from RCM analysis, were subjected to repeated‐measures ANOVA. The pain experienced by the participants was assessed using a paired *t*‐test. Participant satisfaction scores and GAIS scores were scrutinized via the Wilcoxon signed‐rank test.

## 3 Results

### 3.1 Volunteer Information

Among the 32 patients initially enrolled in the study, a total of 30 individuals (24 females and 6 males) successfully completed the research, while two participants were unable to adhere to the follow‐up schedule and therefore withdrew. Among the study participants, 12 individuals had Fitzpatrick skin type III, and 18 individuals had type IV. The average age of the participants was 46.83 ± 6.59 years.

### 3.2 Clinical Manifestation

**FIGURE 1:**
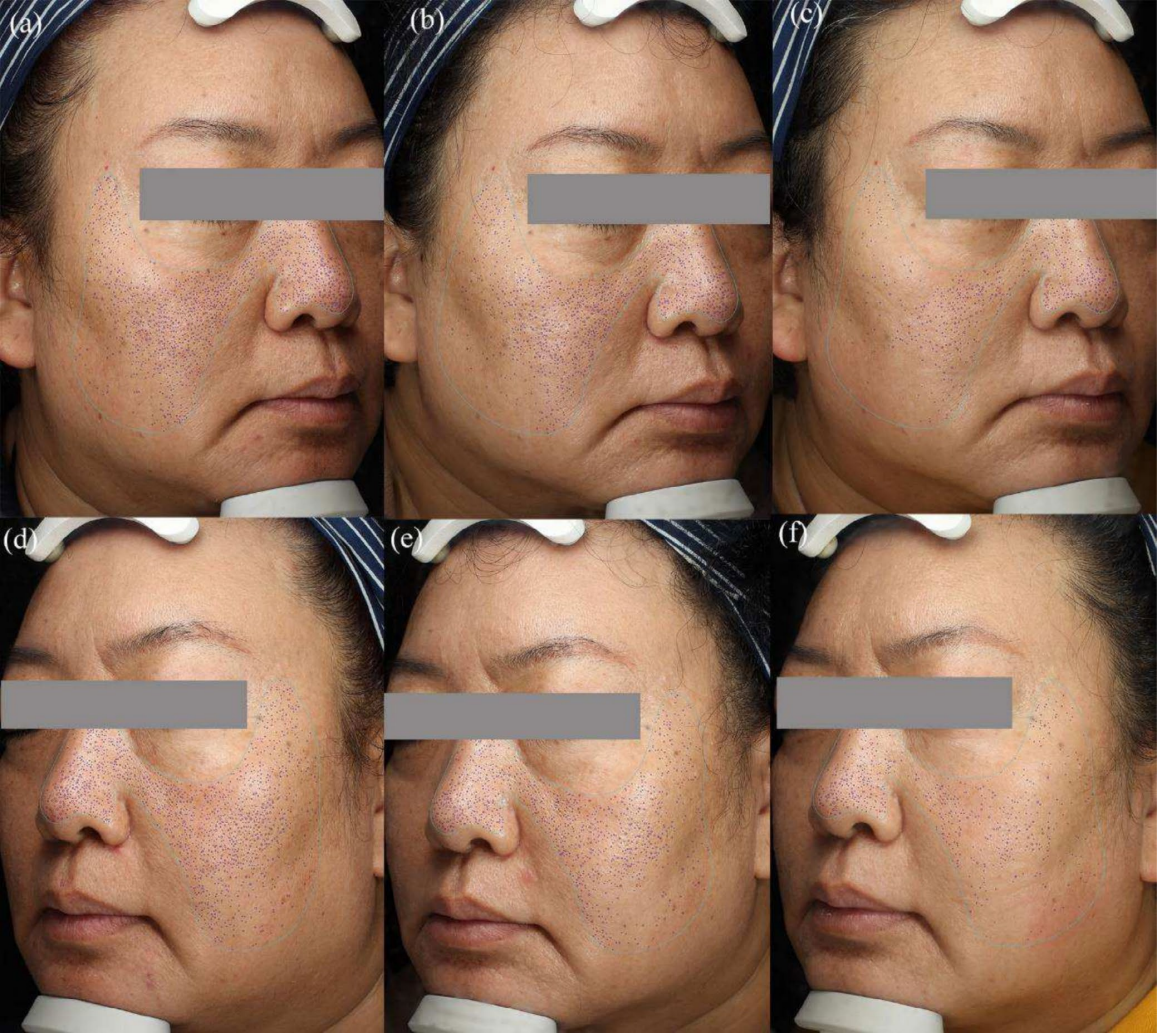
Pore counts images of a 47‐year‐old woman with Fitzpatrick skin type IV. Images were captured at baseline (a), 1 month (b), and 3 months (c) after five treatments with a FxPico laser on the right side of the face. Images were captured at baseline (d), 1 month (e), and 3 months (f) after five treatments with a QSF‐Nd:YAG laser on the left side of the face.

**FIGURE 2:**
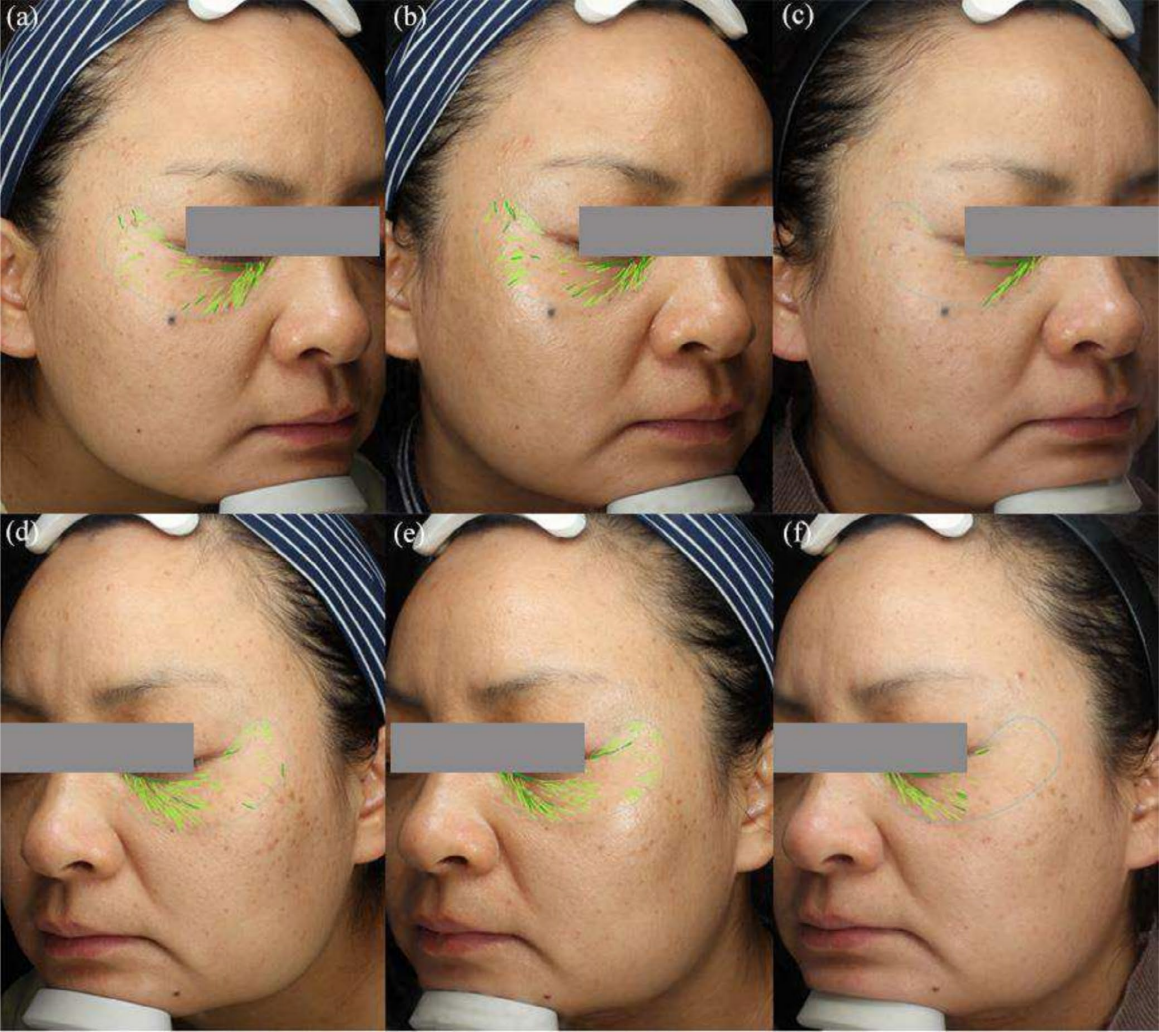
Wrinkles counts and pigment spots on the cheek of a 42‐year‐old woman with Fitzpatrick skin type IV. Images were captured at baseline (a), 1 month (b), and 3 months (c) after five treatments with a FxPico laser on the right side of the face. Images were captured at baseline (d), 1 month (e), and 3 months (f) after five treatments with a QSF‐Nd:YAG laser on the left side of the face.

At the final follow‐up, which took place 3 months after the last treatment, both the FxPico and QSF‐Nd:YAG treatments exhibited notable improvement in visible wrinkles, pore size, and skin texture (Figures 1 and 2). On the FxPico side, the average number of wrinkles decreased from 84.53 ± 25.75 to 52.17 ± 26.11 (*p* < 0.001), while on the QSF‐Nd:YAG side, it decreased from 89.43 ± 25.22 to 50.23 ± 22.18 (*p* < 0.001). The pore counts showed a decrease from 637.73 ± 299.12 to 413.77 ± 231.33 (*p* < 0.001) on the FxPico side and from 647.03 ± 327.32 to 421.03 ± 239.52 (*p* < 0.001) on the QSF‐Nd:YAG side. The average skin texture counts reduced from 1282.50 (849.00, 1795.75) to 795.00 (358.00, 1180.75) (*p* < 0.05) on the FxPico side and from 1367.00 (450.70, 1913.50) to 835.50 (398.50, 1145.75) (*p* < 0.05) on the QSF‐Nd:YAG side. The average semi‐quantitative scores decreased from 13.50 (8.00, 18.00) to 6.00 (4.00, 8.00) (*p* < 0.001) on the FxPico side and from 14.00 (9.75, 16.25) to 6.00 (4.75, 7.25) (*p* < 0.001) on the QSF‐Nd:YAG side.

There were no statistically significant differences observed in the reduction rates of wrinkles (*p* = 0.805), pores (*p* = 0.943), skin texture counts (*p* = 0.942), and semi‐quantitative scores (*p* = 0.662) between the FxPico and QSF‐Nd:YAG sides. Furthermore, the GAIS scores indicated that there were no significant differences in the clinical improvements between the two treatment sides (*p* = 0.234).

### 3.3 Histological Examination

Comparative analysis with baseline measurements revealed an increase in dermal collagen fibers, as visualized under microscopy using picric acid and hematoxylin–eosin staining. Notably, the newly formed collagen fibers exhibited greater density and a more orderly, horizontally oriented arrangement.

**FIGURE 3:**
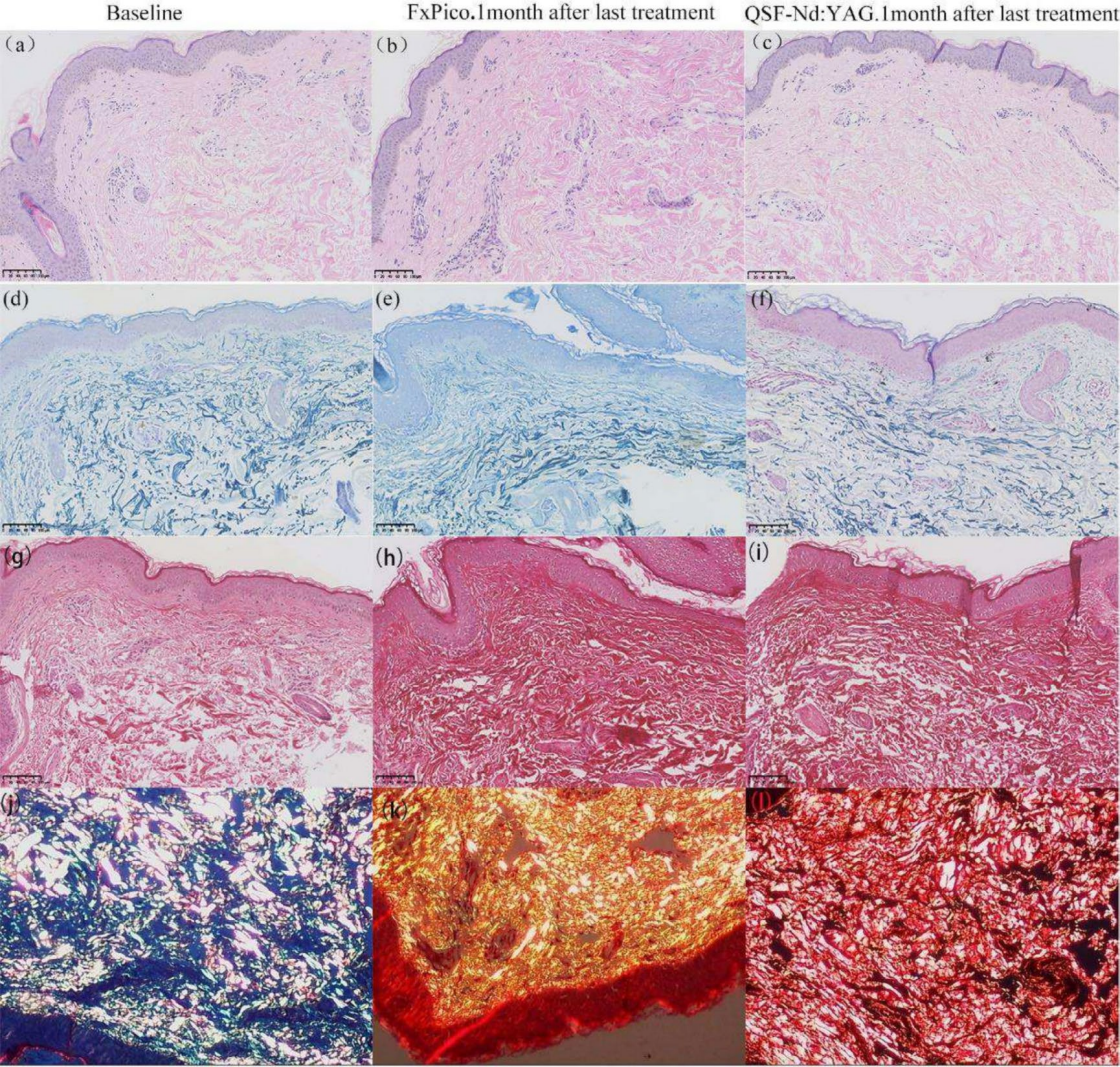
Histological changes at baseline and 1 month after last treatment with FxPico and QSF‐Nd:YAG laser. (a–c) Compared with baseline, the collagen in both groups after treatment was increased and thickened horizontally and arranged in a more orderly manner, with more collagen in the picosecond side than in the nanosecond side, with thicker diameter and denser arrangement. Hematoxylin–eosin; original magnification ×200. (d–f) Histological changes in elastic fibers at baseline and 1 month after last treatment with FxPico and QSF‐Nd:YAG laser. An elongated and more orderly arrangement of the elastic fibers is observed. Elastic (Weigert) staining; original magnification ×200. (g–i) Images captured under a light microscope show new denser and horizontally oriented collagen in the dermis of two sides. (j–l) Images captured under a polarized microscope show an increase in collagen I (red) after treatment, indicating newly synthesized collagen. Picrosirius red; original magnification ×200.

In Figure 3, compared to baseline (a, g), 1 month after the final FxPico and QSF‐Nd:YAG laser treatments (b, h and c, i), collagen fibers demonstrated increased thickness and horizontal alignment, along with improved structural organization. Upon closer examination, the FxPico‐treated area showed a more pronounced trend in collagen proliferation than the QSF‐Nd:YAG‐treated region, though this difference did not reach statistical significance.

At baseline (Figure 3d), elastic fibers appeared fragmented, twisted, and disorganized, whereas 1 month post‐treatment (Figure 3e,f), they exhibited enhanced elongation and alignment.

**FIGURE 4:**
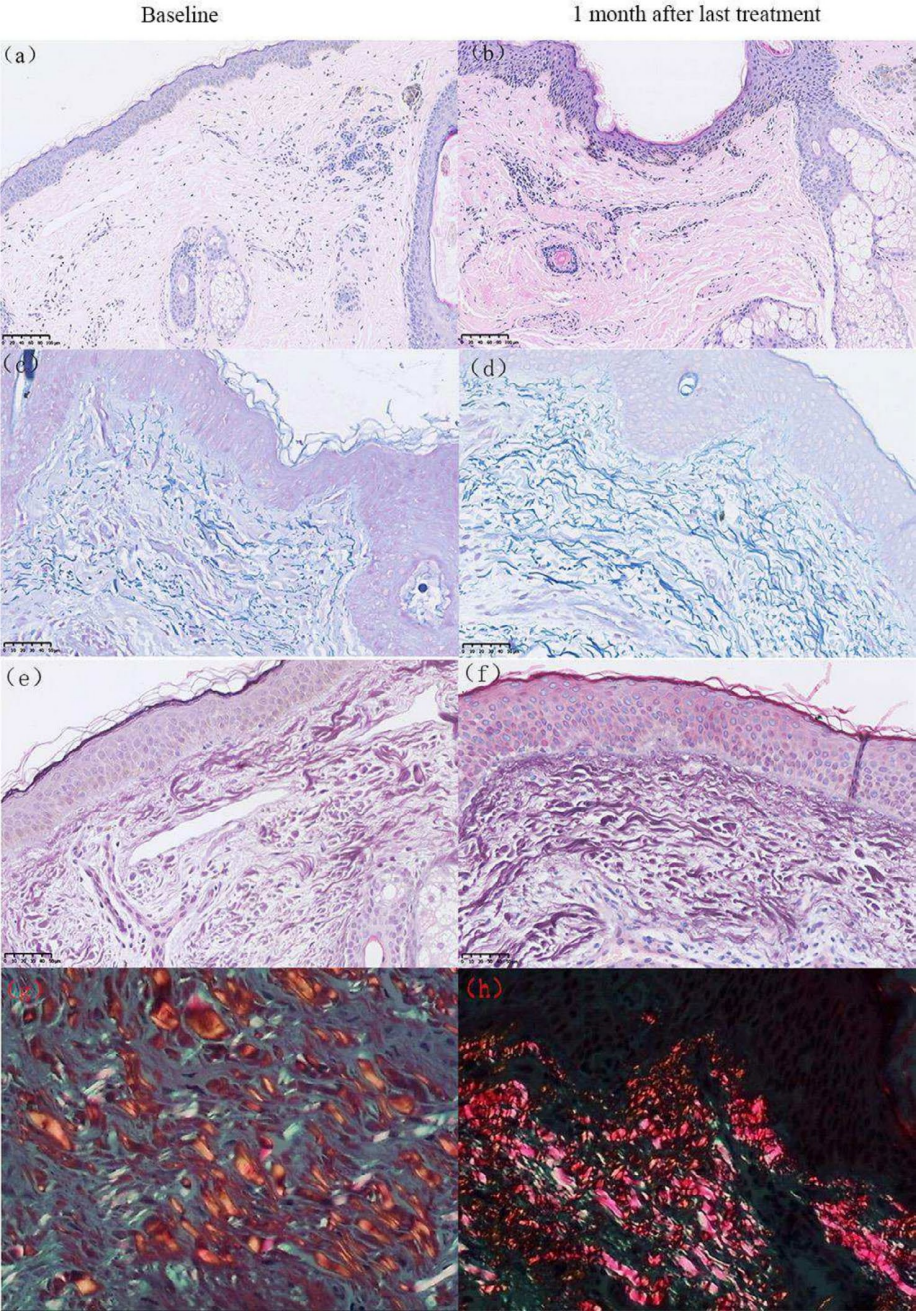
Histological changes at baseline and 1 month after last treatment with QSF‐Nd:YAG laser. (a, b) Compared to baseline, new denser and horizontally oriented collagen in the dermis of 1 month after last treatment is observed. Hematoxylin–eosin; original magnification ×200. (c, d) Histological changes in elastic fibers at baseline and 1 month after last treatment with QSF‐Nd:YAG laser. An elongated and more orderly arrangement of the elastic fibers is observed. Elastic (Weigert) staining; original magnification ×400. (e, f) Images captured under a light microscope show new denser and horizontally oriented collagen in the dermis of two sides. (g, h) Images captured under a polarized microscope show an increase in collagen I (red) after treatment, indicating newly synthesized collagen. Picrosirius red; original magnification ×400.

In the second histological assessment (Figure 4), compared to baseline (a, e), 1 month after the final QSF‐Nd:YAG treatment (b, f), the dermis displayed newly formed, densely packed collagen fibers with a horizontal orientation. Similarly, while elastic fibers at baseline (c) were irregular and fragmented, post‐treatment observations (d) revealed increased length, improved organization, and greater abundance.

**FIGURE 5:**
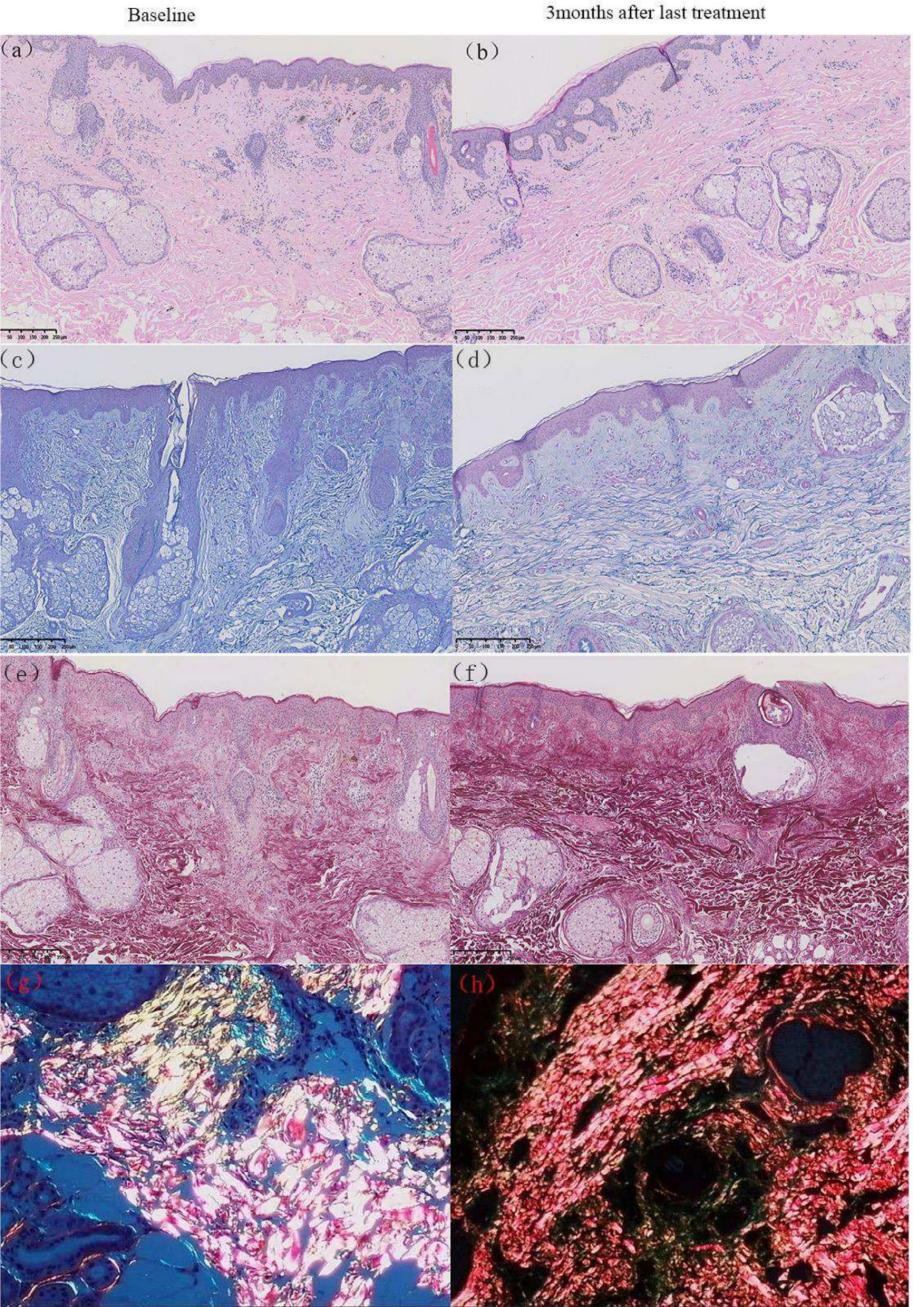
Histological changes at baseline and 3 months after last treatment with FxPico laser. (a, b) An increased, thickened and more ordered alignment of collagen levels was observed. Hematoxylin–eosin; original magnification ×100. (c, d) Histological changes in elastic fibers at baseline and 3 months after last treatment with FxPico laser. An elongated and more orderly arrangement of the elastic fibers is observed after treatment. Elastic (Weigert) staining; original magnification ×400. (e, f) Images captured under a light microscope show new denser and horizontally oriented collagen in the dermis of 3 months after last treatment. (g, h), images captured under a polarized microscope show an increase in collagen I (red) after treatment, indicating newly synthesized collagen. Picrosirius red; original magnification ×400.

In the third histological examination (Figure 5), compared to baseline (a, e), 3 months after the final FxPico treatment (b, f) the dermis exhibited new, denser and horizontally aligned collagen fibers. Elastic fibers, which appeared disordered and broken at baseline (c), demonstrated prolonged structural improvement (d), including increased length, enhanced alignment, and greater quantity, underscoring the sustained efficacy of FxPico laser treatment.

Under polarized light microscopy, picric acid‐stained collagen exhibited distinct color profiles: type I collagen (newly synthesized) appeared red and yellow, while type III collagen (smaller fibers) stained green. Compared to baseline (Figures 3j, 4g and 5g), post‐treatment samples (Figures 3k,l, 4h and 5h) showed a significant increase in Type I collagen abundance, further confirming the role of both treatments in stimulating neocollagenesis.

### 3.4 Adverse Events

Both treatment modalities demonstrated excellent safety profiles, as adverse events were mild in nature and self‐limiting. The FxPico‐treated area, observed adverse effects included erythema (lasting for an average of 3.70 ± 1.19 days) and petechiae (lasting for an average of 4.34 ± 1.24 days). Conversely, the QSF‐Nd:YAG‐treated area experienced pinpoint bleeding (lasting for an average of 4.36 ± 1.44 days) and slight itching, which subsided within approximately 1–3 days. Statistical analysis revealed no significant differences in the duration of erythema (*p* = 0.184) or in the resolution of pinpoint bleeding or petechiae (*p* = 0.800) between the two treatment areas. Notably, the average pain Visual Analog Scale (VAS) score for the QSF‐Nd:YAG‐treated area was significantly lower (1.48 ± 1.35) compared to the FxPico‐treated area (2.84 ± 0.98) (*p* < 0.0001). It is worth mentioning that two subjects with a history of recurrent herpes simplex virus (HSV) experienced a reoccurrence of symptoms post‐treatment. One patient experienced this phenomenon after receiving treatment on both sides, while another patient experienced it solely in the picosecond laser group. Fortunately, all cases recovered following standard antiviral therapy, and no permanent side effects were observed. All participants expressed a certain level of satisfaction with both treatments, and there were no significant differences in participant satisfaction observed between the two groups (*Z* = 0.00, *p* = 1.00).

## 4 Discussion

Photoaging, also known as extrinsic aging, primarily arises due to the detrimental effects of ultraviolet (UV) radiation. It manifests as various dermatological manifestations, including pigment irregularities, profound wrinkles, erythema, telangiectasia, alterations in skin texture, and enlarged pores. A diverse array of therapeutic approaches have emerged as potential remedies for ameliorating photoaging. Nonetheless, the widespread adoption of these interventions is hindered by their suboptimal efficacy and excessive adverse effects. Ablative laser therapy, while effective in treating severe photoaging, inflicts direct damage to the epidermis, giving rise to noticeable pain during the procedure and protracted postoperative recovery periods. Additional undesired consequences include post‐inflammatory pigmentation, infection, and a heightened risk of post‐inflammatory pigmentation, particularly among individuals with darker skin tones [5]. The administration of topical retinoids and adapalene gel has been associated with adverse effects such as burning sensations, erythema, peeling, itching, and dryness, further detracting from their overall appeal as treatment options [12, 13].

The Q‐switched fractional 1064‐nm Nd:YAG laser is distinguished by its abbreviated pulse duration, imparting a photomechanical effect upon the target tissue. Its elongated wavelength facilitates deep penetration into the underlying layers. Manipulation of the laser beam is achieved through employment of a diffractive lens, enabling the delivery of multiple microscopic laser beams to specific treatment zones while sparing the intervening areas. This judicious approach results in minimal epidermal damage and a shorter overall recovery period. Consequently, intra‐dermal disintegration, coagulation, and damage are elicited, thereby initiating the wound‐healing response that prompts collagen synthesis and stimulates dermal remodeling [5, 14]. Previous investigations have ascertained that the primary culprits behind skin aging are the aberrant expression of matrix metallopeptidase (MMP) and tissue inhibitor of metalloproteinase (TIMP) in the skin. MMPs are major contributors to tissue remodeling and collagen degradation, which expression is increased instigating the degradation of the dermal extracellular matrix, specifically procollagens I and III.

The Q‐switched fractional 1064 nm Nd:YAG laser modulates the expression levels of collagen, TIMP, and MMP by downregulating miR‐24‐3p, thereby fostering collagen synthesis and fortifying the protective effects on the skin barrier [15]. Moreover, it activates ERK1/2 and P38MAPK signaling pathways [16, 17]. Roh et al. [18] conducted an investigation into the efficacy of the Q‐switched Nd:YAG laser in 10 patients with dilated pores, employing a split‐face study design. The findings revealed a notable improvement in the reduction of enlarged pores. Likewise, Urdiales‐Gálvez et al. [14] reported marked to excellent improvements in face and neck rejuvenation in 16 women following non‐ablative fractional 1064‐nm Q‐switched Nd:YAG laser treatment. Similarly, Akerman et al. [19] explored the effects of QSF‐Nd:YAG laser therapy in 11 patients with significant scarring, observing a stimulating effect on collagen production and dermal remodeling.

The picosecond laser, characterized by its short pulse duration, offers a novel non‐ablative method for skin repair. This innovative technology harnesses the power of photomechanical effects over undesirable photothermal effects, generating precise micro‐injury zones in the epidermis and dermis [20, 21]. Histologically, these micro‐injury zones manifest as LIOB intraepidermally and laser‐induced cavitation (LIC) intradermically. This will initiate wound repair and trigger the secretion of cytokines and chemokines from keratinocytes, stimulating the production of new collagen, elastin, and mucin for dermal remodeling [20, 21].

In an in vitro skin experiment, Yeh et al. demonstrated that low‐energy picosecond laser pulses can penetrate deeply into the dermis, inducing LIC and promoting collagen regeneration. Conversely, high‐energy pulses primarily cause LIOB in the epidermis [22]. However, it is important to note that this particular study was conducted on skin samples in vitro. Our study was conducted on in vivo tissue using energy levels of 1.9–2.1 mJ/microbeam. As shown in Figure 3, histopathological analysis confirmed collagen augmentation. Nevertheless, these findings do not demonstrate that the deeper and more extensive intradermal LICs have superior rejuvenation effects compared to the more superficial intra‐epidermal LIOBs. Wu et al. conducted a study comparing the efficacy of fractionated frequency‐doubled 1064/532 nm picosecond Nd:YAG laser and fractionated 1927 nm thulium fiber laser for half‐face photo rejuvenation. Both treatment groups exhibited significant improvements in elastic tissue, erythematosis, keratosis, pigmentation abnormalities, and skin texture. Notably, the picosecond laser demonstrated shorter healing times compared to the other laser modality [8]. Similarly, our study observed short healing times following picosecond laser treatments. Our data align well with their reported results. Our findings corroborate and extend the work of Lee et al. [23] concluded that fractional 1064‐nm picosecond laser is a safe and effective therapeutic option for skin rejuvenation, showcasing significant improvements in skin wrinkles, texture, depressions, and pores. The split‐face controlled design employed in this study, coupled with histological confirmation, provides robust evidence. Additionally, Palawisuth et al. [24] evaluated the long‐term efficacy and safety of a 1064‐nm picosecond laser with a fractionated microlens array in the treatment of enlarged pores in Asians, reporting significant improvements in pore size reduction. Yim et al. who conducted a split‐face comparative study to evaluate the efficacy of picosecond 1064‐nm Nd:YAG laser versus quasi‐long pulsed 1064‐nm Nd:YAG laser for treating facial photoaging wrinkles and pores. Five treatments were administered at 2‐week intervals, with subjective scale assessments were performed at 10 weeks post‐treatment. Results demonstrated that both devices effectively improved wrinkles and pore appearance, with comparable efficacy in treating photoaging‐related wrinkles and enlarged pores [3]. The results obtained in the present study are in close agreement with previous reports by Palawisuth et al. and Yim et al. further validating the therapeutic efficacy of both laser modalities for photoaging.

At the conclusion of the treatment sessions, both the FxPico laser and the QSF‐Nd:YAG laser demonstrated efficacy in addressing photoaging facial wrinkles and pores. After undergoing five treatments, an overall improvement in facial photoaging was observed. However, no statistically significant differences were found in the degree of improvement in photoaging between the two laser modalities, suggesting that both lasers were equally effective in enhancing photoaging wrinkles and reducing enlarged pores. This equivalence in efficacy can be attributed to their respective mechanisms of action. Both modalities generate micro‐injury zones within the skin, initiating the wound repair procedure. The resulting healing involves the formation of new collagen and elastic fibers, aligning with the histological findings of this study.

Histological examinations conducted at 1 and 3 months after the completion of five treatments were performed to evaluate the long‐term effects of both lasers. These examinations revealed the production of new collagen in the upper dermis layer following both laser treatments, with slender and orderly elastic fibers, consistent with the findings reported by Zhang et al. [1]. Adult skin often contains a higher proportion of type I collagen fibers, and our results are the same as the facts. From Figure 3g–i, it is evident that picosecond fractional laser treatment resulted in a higher number of collagen hyperplasia, thicker collagen fiber diameters, denser arrangements of elastic fibers, and a more orderly appearance. We speculate that the picosecond fractional 1064 nm Nd:YAG laser may stimulate greater collagen fiber proliferation compared to the Q‐switched fractional 1064 nm Nd:YAG laser. Nevertheless, this observation remains purely descriptive and lacks statistical significance. Further studies are needed to confirm these findings.

In this study, reflectance confocal microscopy (RCM) was implemented to semi‐quantitatively assess photoaging, thereby providing more objective and scientific data. RCM revealed a regular arrangement of collagen fibers in the dermis layer, with a greater prevalence of thick collagen fibers, corroborating the histological findings.

Although statistically significant pain was reported following FxPico treatment, both treatment modalities were well tolerated, and no serious adverse events occurred throughout the duration of this trial. Following treatment, both groups exhibited temporary mild to moderate degrees of erythema, petechiae, and punctate hemorrhage, which voluntarily subsided after a few days. The limited side effects can be attributed to the micro‐injury zones created by the lasers, where the surrounding tissue remains unaffected, allowing for rapid epidermal repair through the migration of viable cells. Consequently, downtime is minimal, and the skin barrier remains relatively undamaged [25].

In this research, a subset of participants developed mild adverse events, including erythema, petechiae, pruritus, pain, and HSV reactivation. These complications were successfully managed through immediate postoperative cryotherapy to minimize erythema and petechiae formation, oral antihistamine therapy for pruritus control, and oral nonsteroidal anti‐inflammatory drugs (NSAIDs) for pain relief. Notably, two individuals with a history of recurrent HSV were found to experience reactivation after treatment. While reports of HSV reinfection induced by fractional ablative laser have been documented [26], occurrences with non‐ablative lasers are rare. This suggests that laser therapy may trigger reinfection in patients with a history of recurrent HSV, emphasizing the need for prophylactic antiviral therapy when administering laser treatments [27]. Researchers recommend initiating antiviral prophylaxis (valacyclovir 500 mg twice daily) 1 day prior to fractional non‐ablative treatment and continuing for 14 days in patients with a history of facial HSV infection.

Both the FxPico laser and the QSF‐Nd:YAG laser therapies show comparable efficacy in wrinkle reduction and pore improvement with good tolerability. Nevertheless, QSF‐Nd:YAG laser treatment provides superior cost‐effectiveness, making it a more economically feasible option for patients pursuing equivalent aesthetic results.

Several limitations should be acknowledged in this study: firstly, a larger participant sample is necessary to explore age stratification; secondly, the follow‐up duration was relatively short; and thirdly, this study employed a half‐face controlled trial design, potentially exposing the systemic effects of local trauma, such as inflammatory responses, which may influence the study outcomes. Therefore, the inclusion of a control group is warranted to further elucidate the distinctions between the two treatments.

## 5 Conclusion

This study demonstrates the effectiveness and safety of both the fractional 1064 nm Nd:YAG picosecond laser and the fractional Q‐switched 1064 nm Nd:YAG laser as treatment modalities for improving photoaging.

## Author Contributions

Qiuyue Tang and Yuhong Xie performed the research. Qingbiao Wa and Qilei Che supervised the research study. Qiuyue Tang, Yulian Gao, Lufeng Liu, and Zixuan Zhou analyzed the data. Qiuyue Tang and Qilei Che wrote the paper. Pengyu Zhao, Qiang He, and Lixia Xie performed the skin biopsy. Wenju Wang directed the RCM operation. All authors have read and agreed to the published version of the manuscript.

## Ethics Statement

The present participants in this project were adhered to all Helsinki ethical principles. The study protocol received approval from the Institutional Review Board of the Chengdu Second People's Hospital (Approval No: 2022056).

## Consent

Permission was obtained for publishing clinical pictures by the patients.

## Conflicts of Interest

The authors declare no conflicts of interest.

## Data Availability

The data that support the findings of this study are available on request from the corresponding author. The data are not publicly available due to privacy or ethical restrictions.
